# File notes and breach reports: when are they appropriate?

**DOI:** 10.1186/1745-6215-14-S1-P133

**Published:** 2013-11-29

**Authors:** Alison McDonald, Seonaidh Cotton, John Norrie

**Affiliations:** 1 University of Aberdeen, Aberdeen, UK

## 

Protocol and/or GCP deviations occur in many clinical trials. There are clear definitions and reporting guidelines on serious breaches in Clinical Trials of Investigational Medicinal Products (CTIMPs)[1]. However there is no such national guidance for non-CTIMPs. For any trial design, there is little, if any, information on when and for what type of incident it is appropriate to write a file note or when a breach report is the appropriate action. Should we consider whether an event that has been recorded by a file note but then repeated becomes a breach? There is also lack of clarity about the jargon we (and others) use - violation, deviation, breach - do they all mean the same or, if not, which is the most appropriate terminology to use for what type of event? This lack of clarity leads to confusion and inconsistency.

We will present incidents that we have been directly involved in and discuss how we have handled these. We will also discuss how we have developed our guidance on recording file notes and breaches. This will include definitions, information recorded, how decisions are made and by whom and when oversight by sponsor is required.
